# Structural Properties of Human IAPP Dimer in Membrane Environment Studied by All-Atom Molecular Dynamics Simulations

**DOI:** 10.1038/s41598-017-08504-x

**Published:** 2017-08-11

**Authors:** Na Liu, Mojie Duan, Minghui Yang

**Affiliations:** 1 Key Laboratory of Magnetic Resonance in Biological Systems, National Center for Magnetic Resonance in Wuhan, State Key laboratory of magnetic Resonance and Atomic and Molecular Physics, Wuhan Institute of physics and Mathematics, Chinese Academy of Sciences, Wuhan, 430071 China; 2 University of Chinese Academy of Sciences, Beijing, 100049 People’s Republic of China

## Abstract

The aggregation of human islet amyloid polypeptide (hIAPP) can damage the membrane of the β-cells in the pancreatic islets and induce type 2 diabetes (T2D). Growing evidences indicated that the major toxic species are small oligomers of IAPP. Due to the fast aggregation nature, it is hard to characterize the structures of IAPP oligomers by experiments, especially in the complex membrane environment. On the other side, molecular dynamics simulation can provide atomic details of the structure and dynamics of the aggregation of IAPP. In this study, all-atom bias-exchange metadynamics (BE-Meta) and unbiased molecular dynamics simulations were employed to study the structural properties of IAPP dimer in the membranes environments. A number of intermediates, including α-helical states, β-sheet states, and fully disordered states, are identified. The formation of N-terminal β-sheet structure is prior to the C-terminal β-sheet structure towards the final fibril-like structures. The α-helical intermediates have lower propensity in the dimeric hIAPP and are off-pathway intermediates. The simulations also demonstrate that the β-sheet intermediates induce more perturbation on the membrane than the α-helical and disordered states and thus pose higher disruption ability.

## Introduction

The misfolding and aberrant aggregation of proteins or peptides into oligomers and mature amyloid are believed to be the pathology of more than 50 human diseases, including Alzheimer’s disease (AD), Parkinson disease (PD), Huntington disease (HD) and type-2 diabetes (T2D)^1^. Although the sequences of the amyloid proteins are distinct from each other, the amyloid deposits commonly share the so-called “cross-β” structures^2, 3^. It is great important to understand the molecular mechanisms of these soluble molecules aggregating to the β-sheet-rich fibrils.

The amyloidosis of type-2 diabetes is related to a 37-residue peptide hormone, human islet amyloid plolypeptide (hIAPP) or amylin^4, 5^. The fibril deposits of hIAPP have been observed in more than 90% individuals with T2D^6^. hIAPP is co-secreted with insulin from the pancreatic β-cells and plays important roles in the regulation of satiety, gastric emptying, and control of glucose and lactate homeostasis^7, 8^. There is a transient α-helix located on the N-terminal segment 8–22^9, 10^ while the segment from residues 20 to 29 are called “amyloidogenic core”^11^. The amyloid deposits of IAPP were usually accompanied with the death of β-cell and the failure of islet cell transplantation^12^.

Growing evidences have demonstrated that the prefibrillar oligomers rather than the mature fibrils are the main toxic species for many amyloidosis peptides^13, 14^ including IAPP^15–19^. Amyloid oligomers for different proteins are different in sizes, structures, and topologies^20^. The IAPP dimer has been proven to be the most probable oligomer at low concentrations^21–26^. The study of structure properties of IAPP oligomers, especially dimers would greatly increase the understanding of the aggregation mechanism of IAPP. However, due to their transient survival nature, it is still very difficult to experimentally characterize the structure and morphology of IAPP oligomers.

Understanding the interactions between cell membranes and amyloidosis proteins is another difficult task. Cell membranes are crucial in the response of IAPP toxicity^12, 27^. Several theories have been proposed to explain the membrane disruption mechanisms induced by IAPP^22, 27–32^. The pore formation model suggests that the hIAPP oligomers act as ion channels or pores, which cause non-physiological leakage of ions or molecules across the membrane. The detergent-like model supposed that the growth of protofibrils inside the membrane or on the membrane surface disturb the membrane integrity, further cause the membrane thinning and fragmentation. Besides, α-helical oligomers were proposed to be on-pathway toxic intermediates in the fibril formation, and membranes promote the formation of the α-helical structures^32–34^. Therefore, realization of the assembly mechanism of hIAPP on membrane environment and the interactions between the early stages oligomers with the membranes is very important to understand the toxicology of IAPP.

Extensive studies, both experimentally^23, 32, 35–43^ and computationally^44–51^, have investigated the structure properties of IAPP monomer, oligomers and assembly of fibrils in the membrane environments. However, detailed mechanism and the structure properties of the early stage of IAPP aggregation process is unclear. In this study, the bias-exchange metadynamics (BE-Meta)^52^, as well as the unbiased molecular dynamics simulations, were employed to study the structure properties of hIAPP dimer in the lipid bilayer. Our simulations uncovered many intermediates of hIAPP dimer in the membrane environment. As a result, the β-strand rich intermediates are more abundant than the α-helical intermediates and the α-helical intermediates are off-pathway states in the assembly process to the amyloid fibers. Moreover, the β-sheet rich intermediates are found to pose higher disruption ability to the lipid bilayers and these toxic oligomers could be potential targets in the development of novel drugs to treat T2D.

## Materials and Methods

### System Setup

The bilayer membranes used in this work are composed by 1,2-dioleoyl-sn-glycero-3-phosphocholine (DOPC) and 1,2-dioleoyl-sn-glycero-3-phospho-L-serine (DOPS). The total number of lipids is 160 and the ratio of DOPC to DOPS is 7:3 (112 DOPC and 48 DOPS), which is consistent with the observed ratio of neutral to anionic phospholipids in β-cell membranes. hIAPP is consisted by 37 amino acids, its sequence is KCNTATCATQ^10^RLANFLVHSS^20^NNFGAILSST^30^NVGSNTY. The peptide was built with an amidated C-terminus and a disulfide bridge between Cys2 and Cys7 in our simulations, which are both required for the biological activity of hIAPP in physiological conditions^53^. The initial structure of hIAPP dimer was extracted from the NMR pentamer structure model^54^. The initially orientation and position of hIAPP dimer in the membranes were set according to the SFG spectrum results^40^, i.e. the central U-shaped regions were immersed into the membrane and the terminus were outside the membrane surface, the initial tilt angle between the β-strand of residue 8 to 16 was set to be 48°, residues R11 and S28 were located on the membrane-water interface. The initial model of IAPP dimer on the membrane was given in Figure S1 and the explanations of the initial model were given in supporting information. The hIAPP insertion was implemented by g_membed module^55^ in GROMACS. After the insertion, 4 DOPC and 2 DOPS were removed from the top leaflet of the bilayer. Then, a 100-ns unbiased MD simulation was carried out to equilibrate the system.

### Bias-Exchange Metadynamics

Bias-exchange metadynamics (BE-Meta)^56^ was carried out to characterize the assembly process and properties of hIAPP dimers. BE-Meta combined the ideas of replica exchange and metadynamics^57^. For metadynamics, the system can escape the energy minima quickly by accumulating history-dependent Gaussian potential on specific collective variables (CVs). However, it is very hard to reach convergence for the systems with more than three CVs. BE-Meta allow conformations exchanged between CVs. In this way, the system can cross the energetic barriers in a short simulation time. In our simulation, eight replicas were employed to explore the structure properties of hIAPP dimer, where two replicas were unbiased but allowed to exchange conformations with the others (the *neutral replicas*). No history-dependent bias potential was added to the neutral replica. Sampling on the neutral replica would provide an approximated canonical distribution when the simulation reach equilibration^52^. Six biased replicas run along six different collective variables, which means one replica serves one CV only. The six biased CVs include: *Q parameter* for residues 8 to 16 and residues 27–35 (CV1), *β-score* for residues 8 to 16 (CV2), *β-score* for residues 27 to 35 (CV3), *β-score* for residues 20 to 29 (CV4), *α-score* for residues 8 to 28 in chain 1 (CV5) and *α-score* for residues 8 to 28 in chain 2 (CV6). Gaussian potential height *w* adopted in this work was 1 *kJ/mol*, bias potential deposited every 5 ps, Gaussian width was 0.4 for CV1 to CV4, 1 for CV5 and CV6, exchange of configurations in different replicas was attemped every 50 ps. The interval range for the six biased CVs are 0–16, 0–9, 0–9, 0–10, 0–18 and 0–18, respectively. In our simulations, the average accptance frequency between the CVs is about 0.13. The exchanges between 8 replicas along the simulation time were given in Figure S3. The detailed description of the biased CVs is given in the supporting information.

### Simulation Details

All simulations were performed with GROMACS5.0.4^58^ patched with PLUMED 2.1.2 plug-in^59^. hIAPP dimer was simulated with amber99SB-ILDN force field^60^, lipids were described with Slipids^61^. TIP3P water model^62^ was used for 9038 water molecules in a box of 75 × 73 × 87 Å^3^. 46 Na^+^ and 6 Cl^−^ were added to neutralize the charged system. The particle-mesh Ewald method^63^ was employed to calculate long-range electrostatics with a cutoff of 12 Å. The same cutoff was used for the Lennard-Jones interactions. Temperature was kept to 300 K by Nosé-Hoover thermostat^64^ with the relaxation time of 0.5 ps. Parrinello-Rahman barostat^65^ was employed to maintain constant pressure of 1.0 bar in NPT ensemble with a characteristic frequency of 2.0 ps. A semi-isotropic scheme was utilized to couple the lateral and perpendicular pressures separately. All bonds length were constrained with LINCS algorithm^66^. A 600 ns simulation was performed for each replica, and the total simulation time was 4.8 μs. The simulations were converged at about 300 ns, and the trajectories after 300 ns were utilized in the following analysis.

### Free Energy Reconstruction

After reaching the covergence at equilibration time t_eq_, the accumulating biased potential *V*_*G*_ in each replica would fluctuate around an average profile^67^. The free energy was estimated as the time average of *V*_*G*_. To reconstruct the free energy from all biased replicas, the structures sampled by BE-Meta were first grouped into clusters, and the free energy of these clusters were calculated by weighted histogram analysis method (WHAM). In this work, the cluster analysis and free energy profile construction were performed using METAGUI^68^ program and some own codes.

### Estimation of Observable Properties

The ensemble average value of any observable property *O* can be calculated based on the estimated free energies as:

1 $$ < O > =\sum _{a}{O}_{a}\exp (-\beta {F}_{a})/\sum _{a}\exp (-\beta {F}_{a})$$

where the sums run over all the clusters on the free energy profile. O_α_ is the average value of *O* in the cluster α. The O_α_ can be reliably estimated when the potential energies of conformations in the same cluster are approximately constant if the clusters are small enough. In this work, the ensemble averages of the secondary structure propensities, number of contact residues and distances between two IAPP chains were analyzed.

### Unbiased Molecular Dynamics Simulations

To further understand the dynamics of the amyloid intermediates and their effects on the membranes, we performed several unbiased molecular dynamics simulations. For different kinds of intermediates, two structures were selected randomly in the corresponding clusters and were used as the initial structures for 200 ns unbiased simulations. The same force fields and simulation parameters were empolyed as described above. In this work, twelve intermediates were selected, and the total unbiased simulation time was 2.4 μs. The area per lipid and membrane thickness analysis were performed by GridMAT-MD^69^ and the curvatures of membrane were calculated by g_lomepro^70^.

## Results and Discussion

### Convergence Tests and Free Energy Profiles as A Function of Collective Variables

**Figure 1:**
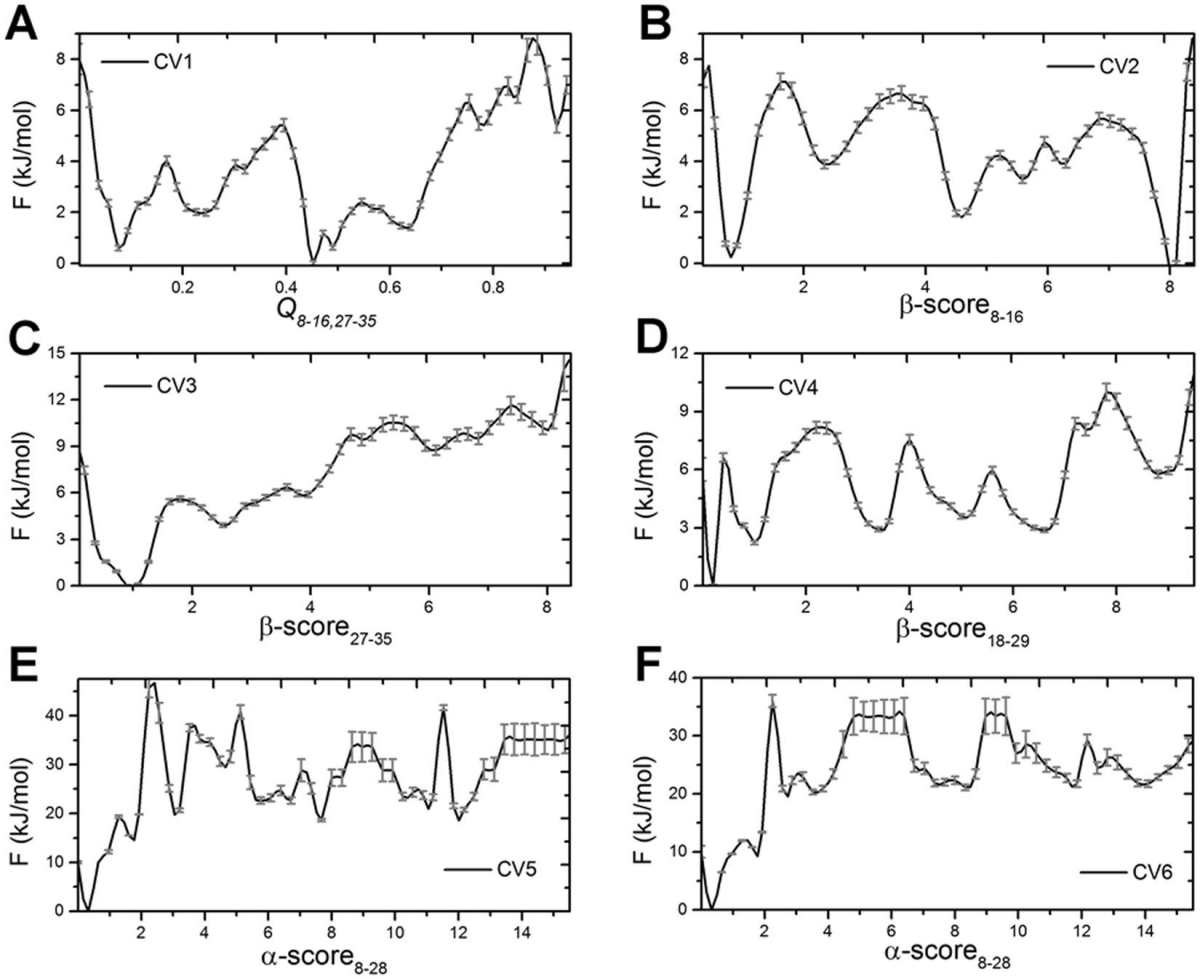
Free energy profiles as function of collective variables. (**A**) Q value for segment 8–16 and 27–35 on the two peptides; (**B**) β-score of segment 8–16; (**C**) β-score of segment 27–35; (**D**) β-score of segment 18–29; (**E**) α-score of segment 8–26 of chain A; (**F**) α-score of segment 8–26 of chain B. The error bars corresponding to the free energy deviation over the last 300 ns of simulations, which were calculated by following the method in ref. 72.

The convergence of BE-Meta simulations was estimated by two criteria. First of all, we calculated the free energy changes as a function of simulation time on each biased collective variables. The free energy changes in a time interval *δt* (*δt* = 20 ns) were given in Figure S4. It can be seen that after 300 ns, the free energy changes in *δt* were less than 4 kJ/mol for all 6 CVs, which is the order of magnitude of thermal fluctuations. Therefore, the convergence of the simulation was achieved at 300 ns. To estimate the free energy deviation after the convergence time, we further calculated the energy fluctuation in the last 300 ns trajectories for each CV. As shown in Fig. 1 the statistical uncertainties of free energy are smaller than 2 kJ/mol for most regions of all 6 CVs, except in the high energy regions of CV5 and CV6 where the uncertainties are around 4 kJ/mol, indicating the convergence in the last part of the simulations. Figure S5 showed the transitions between different intermediates in the last 300 ns of two neutral replicas. The quick transitions between different intermediates further proved the quality of the sampling algorithm.

In the mature fibril of hIAPP, two inter-molecule β-strands are located in the N- and C-terminal regions^54^. The *Q* parameter for the N-terminal region (residue 8–16) and C-terminal region (residue 27–35) was designed to calculate the similarity between the simulated structure and the solid state NMR fibril structure. Larger *Q* value means the simulated structure are closer to the NMR structure. The free energy profile against the Q parameter for residue 8–16 and 27–35 was given in Fig. 1A. The lowest free energy state is located at the position with *Q* value close to 0.5, which indicates that the fibril-like structure is not the most favorable state for the membrane bounded hIAPP dimer. The order parameter β-score evaluates how many residues in the considered segment form ideal β-strands structures^71^. The CV2 to CV4 in this study were utilized to monitor the β-sheet formation in the N-terminal region (residue 8–16), C-terminal region (residue 27–35), and the central region (residue 20–29), respectively. The free energy profiles on these CVs were also shown in Fig. 1. The lowest free energy minimum for CV2 is located at the value close to 8 kJ/mol (Fig. 1B). However, the free energy minimum on CV3 is near 0 kJ/mol, and the free energy of the state with C-terminal strand formed is about 10 kJ/mol (Fig. 1C). The result indicates that the C-terminal is harder to form β-sheet structure. CV5 and CV6 account for the helix structure formation on each chain, it can be seen that the free energy profiles of two helical CVs are similar to each other, and the free energy on the large helix content region are much higher than the small helix content region, which indicate the helical structures is less favorable for the hIAPP dimer.

### Structure properties of IAPP Dimer in Membrane Environments

**Figure 2:**
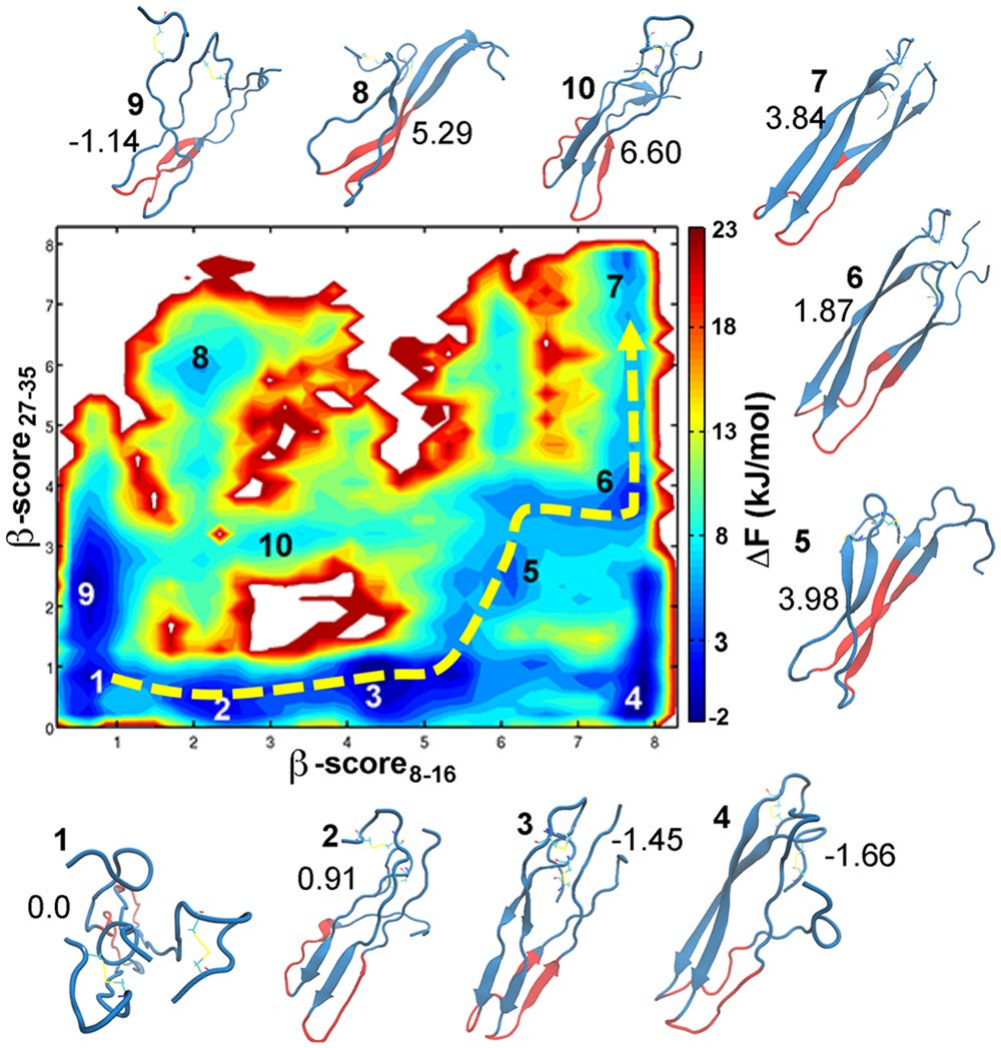
Two dimensional free energy landscape of hIAPP dimer in the membrane environments as the functions of order parameters β-score_8–16_ and β-score_27–35_. The representative structure of free energy minimum on the profile are given, the central region from residue 20 to 29 was colored by red and the disulfide bonds between residues 2 and 7 on each chain were represent in stick model. The yellow dashed line described the dominant free energy pathway from the disordered state to the folded fibril state. The free energy of the minima corresponding to minimum 1 were labeled in the unit of kJ/mol.

To investigate the structure properties of hIAPP dimer, the free energy landscape was constructed against the order parameters of *β-score*_*8–16*_ and *β-score*_*27–35*_ (Fig. 2). As we described above, these two order parameters are corresponding to the β-sheet contents of the N- and C-terminus. The representative structures of free energy intermediates on the landscape were given in Fig. 2. The dominant pathway, which goes from the disordered state (intermediate 1) to the fibril-like state (intermediate 7), was highlighted by a dashed yellow line. The 2D free energy landscape further demonstrated that the fibril-like intermediate is not the most stabilized state of hIAPP dimer in the membrane environments. By using intermediate 1 as the reference, the free energy values of all the intermediates were listed on the figure.

The 20–29 region is believed to play critical role in the fibril formation and toxicity of IAPP. As a homologue of human IAPP, the nontoxic and nonamyloidosis rat IAPP (rIAPP) has only six residues variants with hIAPP on the 20–29 region, and three proline variants in this region are believed to prevent the formation of β-sheet structures and account for the nonamyloidosis of rIAPP. The 20–29 region is often referred as the “amyloidogenic core”. However, the 20–29 region of hIAPP mature fibril is unstructured loop in the solid-state NMR structure. The structure transition process of this region in IAPP aggregation and its role to the aggregation are still ambiguous. Many pioneer work made great efforts to explain the roles of the region. For instance, Buchanan *et al*. employed 2D infrared (2D-IR) spectroscopy and molecular dynamics simulations to study IAPP in aqueous solution and revealed that an intermediate with parallel β-sheet structure in the 20–29 region was presented on the aggregation pathway and act as the lag phase to the fibril formation^11^. The so-called FGAIL intermediate^11^ (FGAIL is the segment 23 to 27 of hIAPP) formed β-sheet before N-terminal or C-terminal strand formed, and ultimately disrupted to form partially disordered loop structure in the fibril.

The FGAIL intermediates were also presented in the assembly of IAPP dimer in the membrane environments. The 20–29 regions were highlighted by red color on the snapshots in Fig. 2. It can be seen that several intermediates have fully or partially β-sheet formed on residue 20–29, such as intermediates 3, 5, and 8. To illustrate that the β-sheet are commonly formed on the structures in these intermediates, we calculated and plot the average β-score_20–29_ as a function of order parameters β-score_8–16_ and β-score_27–35_ (Figure S6). The average β-score_20–29_ are around 8 for intermediates 5, 8 and around 5 for intermediate 3, respectively. Like in the aqueous solution, the FGAIL intermediates (3 and 5) are also present on the minimum free energy pathway from the disordered state 1 to the fibril-like state 7 (the yellow line in Fig. 2). Along the pathway, the fragment 20–29 undergoes disordered-ordered-disordered transition, i.e. it starts from the unstructured loop in intermediates 1 to the fully folded β-sheet structure in intermediates 5, and the β-sheet must break into the partially unstructured loops to form the fibril structure 7.

### N-terminal β-sheet Forms Before C-terminal β-sheet in Membranes

**Figure 3:**
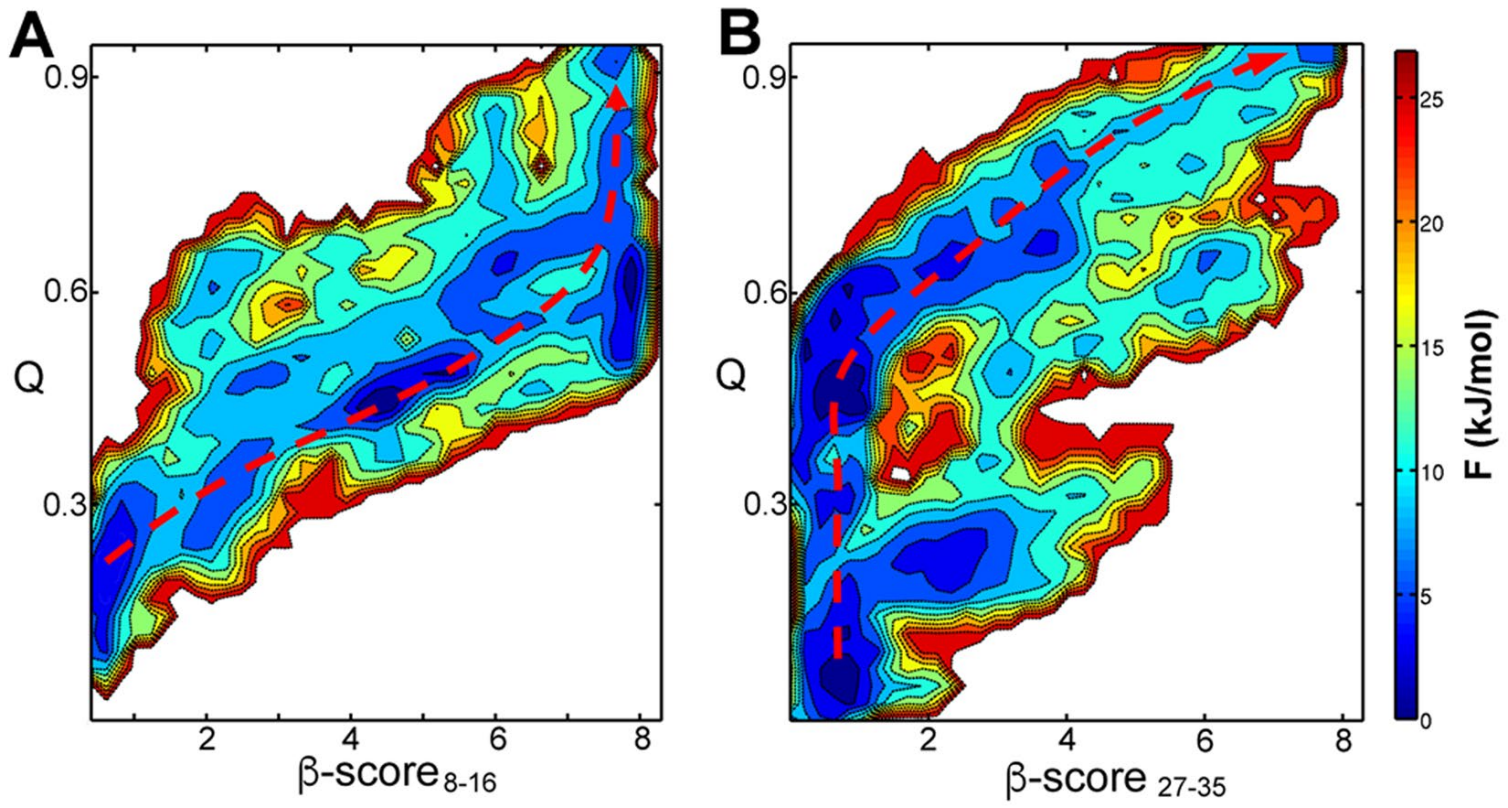
Two-dimensional free energy landscapes of hIAPP dimerization as a function of order parameters β-score_8–16_ and *Q* values (**A**) and β-score_27–35_ and *Q* value (**B**). The red dashed lines represent the lowest free energy pathways from the disordered structures to the fibril-like structures.

Although the FGAIL intermediates were observed in both of the aqueous solution^11^ and the membrane environment, there are some non-trivial different properties of the assembly of hIAPP dimer in different environments. First of all, in the solution, the β-sheet structure on the 20–29 region (FGAIL intermediates) was formed prior to the N-terminal β-sheet formation. And the deformation of the β-sheet structure to unstructured loops was proposed as a rate-limit step in the dimerization process of IAPP. Nevertheless, in the membrane environment the β-sheets on the N-terminal could be simultaneously formed with the central 20–29 region, or even the N-terminal β-sheets can be partially formed prior to the FGAIL intermediates (intermediate 2 in Fig. 2). The roles of FGAIL intermediates are likely to guide or facilitate the formation of C-terminal β-sheets. Secondly, in the aqueous solution there are two comparable pathways from the disordered chains to the fibril-like structures. In one pathway, the N-terminal β-sheet structures were formed firstly. On the contrary, the C-terminal β-sheet structures were formed at first in the other pathway. However, in the membrane environment there is a dominant fibril-like structure formation pathway (The yellow dashed line in Fig. 2). To further analyze the contributions of N- or C- terminal sheets to the fibril formation, we plot the free energy landscapes as function of β-scores and *Q*_*8–16*_,_*27–35*_ parameters (Fig. 3). It can be seen that the growth of N-terminal β-sheet is basically synchronous with the increasing of *Q*_8–16,27–35_ before the value 0.5 (Fig. 3A), and after the fully formation of N-terminal β-sheets, the *Q* value keep going up to 1. However, the *Q* increased to about 0.5 with the β-score_27–35_ close to 0, and after that, the β-scores on the C-terminal region are increased with the *Q* value (Fig. 3B). The results further demonstrate that the N-terminal β-sheets are formed prior to the C-terminus in the assembly process of hIAPP dimer to the fibril structure in membrane environment.

Shim *et al*. employed two-dimensional infrared (2D IR) spectroscopy and isotope labeling technologies to study the kinetic properties of hIAPP aggregation^37^. The fibril structure formation rate on six residues in different regions (residues A8, A13, V17, A25, A27 and V32) were monitored. They revealed that the site 17 in the N-terminal β-strand has the smallest average time to half-maximum value (*t*_*50,ratio*_ = 0.85), which means this site form the parallel-sheet structure firstly. Sites A13 and A25 have comparable *t*_*50,ratio*_ values (1.09 and 1.02, respectively). The C-terminal residue V32 forms the ordered β-sheet structure at the last with the largest *t*_*50,ratio*_ value (1.40). The process of IAPP dimer assembly revealed by our simulations is consistent with the experiment data, i.e. the formation of N-terminal β-strands and the middle region occurs before the formation of C-terminal β-strands in the amyloid fiber formation. Moreover, Dunkelberger *et al*. proved that the deamidation in the N-terminal would lead to the disruption the fiber structures at both the N- and C-terminal β-sheet^73^, which also implied the critical role of N-terminal β-sheet formation in the IAPP aggregation process.

### β-sheet States Have Higher Propensity Than α-helical State for Dimeric hIAPP

**Figure 4:**
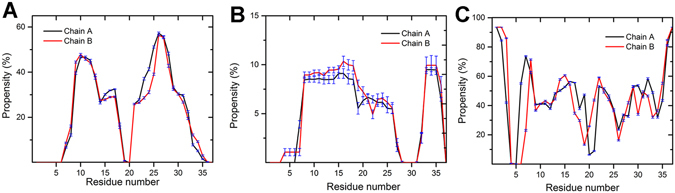
Fraction of secondary structures by residues on each chain. (**A**) β-sheet; (**B**) α-helix; (**C**) random coils. The fractions were reweighted based on Eq. 1. Error bars are calculated from the errors of microstate free energies by following the method described in ref. 72.

To illustrate the secondary structure composition of the dimer hIAPP, the reweighted propensities of per-residue secondary structure on both chains are shown in Fig. 4. Two IAPP chains have similar secondary structure propensities. Similar to the NMR structure of micelle-bound monomeric hIAPP, the helical residues are mainly located in the region 8 to 25^35, 36^ and the helical propensity on the region 8 to 18 is slightly higher than the region 19 to 25. Besides, a short 3_10_-helix structure exist on the C-terminal of the peptides (Fig. 4B), which is also consistent with the experimental results^74^. However, unlike the monomeric hIAPP is prone to form α-helical structure in the membrane environment, hIAPP in the dimeric state has relatively higher β-sheet propensity (~21% β-sheet probability of all residues) than helix (~5.2% helix probability of all residues). The life time for the β-sheet oligomers might be very short, however in the case of abundant oligomers exist, they still can present the toxicity.

The preference of β-sheet structures of dimeric hIAPP found in this study is consistent with many previous experimental studies^11, 75^. For instance, by combining the ion mobility spectrometry and the mass spectrometry (IMS-MS) technology, Bleiholder, *et al*. demonstrated that the extended β-strand conformations are the major classes of small hIAPP oligomers in solution^75^. However, it doesn’t mean that the α-helical hIAPP is absent on the membrane. Actually the membranes would stabilize the α-helical structure of individual monomeric IAPP. The commonly used experimental technologies in the study of dynamic structure properties, such as NMR and CD, only provide an average picture of configuration ensembles in the aggregation process. It is hard to distinguish the oligomers from monomers, the helical structure signal obtained by such experimental data might came from the IAPP monomer, instead of the oligomer intermediates.

Besides, the β-sheet rich oligomers of IAPP in aqueous solution were captured by many computational work by using different force fields and sampling technologies. For instance, the simulation of IAPP dimer in the implicit solution revealed that the β-sheet states have the lowest free energy for human IAPP, on the other hand, the α-helical intermediate is the lowest free energy minima for the non-amyloidosis rat IAPP^25^. A recent computational study of IAPP dimer in the solution which employed REMD technology also indicated the majority of β-sheet structure for hIAPP dimer^76^. Together with the higher propensity of β-sheet structures than α-helical structure in the membrane environments revealed in this study, we speculate the formation of β-sheet rich states is an inherent property of IAPP oligomers, regardless the external environments.

### α-Helical Structures Are Off-pathway States for the hIAPP Dimerization in Membranes

There are two opposite views for the role of α-helical states in the IAPP aggregation process. Based on the higher propensity of the helicity of monomeric hIAPP on membrane and the fact that membrane would promote amylin fibril formation, the α-helical states were hypothesized to be important on-pathway intermediates for the IAPP aggregation^77^. On the other hand, growing evidences show that α-helical structures are unnecessary for the fibril formation and might be off-pathway in the assembly process. A recent study show that the hIAPP with D-residues (D-hIAPP), which could not form α-helical structure, have equivalent kinetics of self-assembly with the wild-type hIAPP^43^. In order to illustrate the role of helical structure dimer in the aggregation, detailed analysis is required.

**Figure 5:**
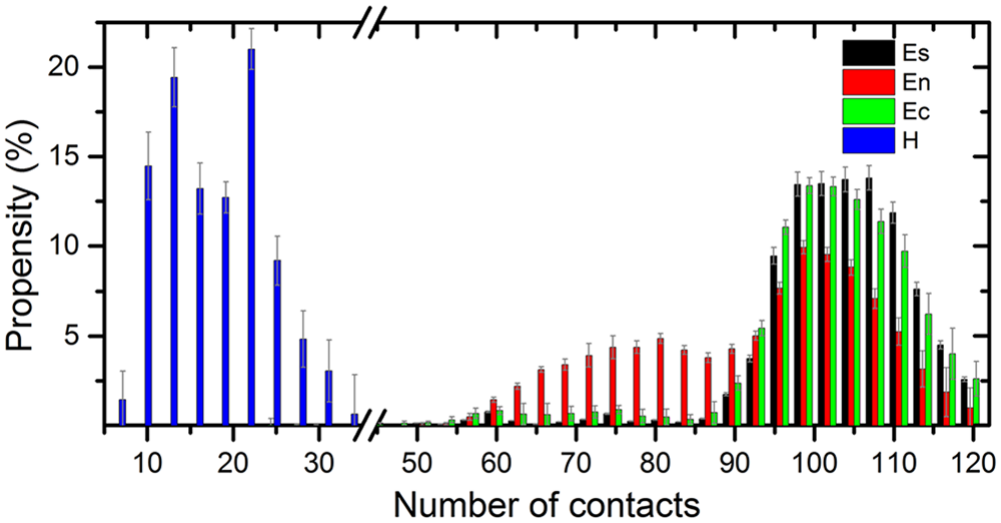
Propensity of inter-chain contact number in different intermediates. Es: intermediate with more than six β-sheet residues on both N-terminal (residue 8–17) and C-terminal region (residue 28–35); En: intermediate with more than six β-sheet residues on the N-terminal region; Ec: intermediate with more than six β-sheet residues on C-terminal region; H: intermediate with more than eight α-helical residues on both chains. Two inter-chain residues were defined to be contact if the distance between any heavy atoms on these residues is less than 4.5 Å. The error bars are corresponding the deviations of the propensity values.

We analyzed the location of α-helical intermediates on the IAPP dimer assembly pathway by plotting the α-score of two chains against the β-score_8–16_ and β-score_27–35_ (Figure S7). By comparing it with the free energy landscape as shown in Fig. 2, it can be seen that the conformations with α-helix structure (large α-scores) are basically located in the left bottom region in which both the β-score_8–16_ and β-score_27–35_ are close to 0 (Blue region in Figure S7). These structures are absent from the dominant path of fibril structure formation of hIAPP, indicating that the α-helical states are the off-pathway intermediates. To characterize the interaction between two IAPP chains in different intermediates, we calculated the average contact residue number and distances between the two chains in the conformations with different kinds of secondary structure compositions (Fig. 5 and Figure S8). As shown in Fig. 5, the contact residue numbers in the intermediates with long β-sheet fragments are significantly larger than that in the intermediates with long α-helical structures. Moreover, the distribution of two chain distances in different intermediates also showed that the two peptides are separated in the structures with α-helix structure formed on any of the chain. The small contact residue number and large distance in the α-helical intermediates indicate that it is unlikely to form a compact α-helical structure for hIAPP dimer in the membrane environments.

Although the anionic membranes are able to stabilize the α-helical structure of monomeric IAPP, it lacks direct observations to demonstrate the helical hIAPP oligomers exist on the fibril formation pathways. On the other side, Carufel *et al*. proved that the inhibition of helical formation greatly facilitates the self-assembly of anionic bio-surface bound hIAPP. Moreover, the restrain of α-helical structure would accelerate the leakage of model membranes^43^. A recent computational work also implied the irrelevance of α-helical structures to the IAPP aggregation, the structure ensemble analysis showed that the aggregation inhibitor EGCC would increase the propensity of α-helical structures and decrease the propensity of β-sheet rich structures^76^. Our study further demonstrate the α-helical intermediates are off-pathway in the amyloid fibril formation in the membrane environments.

### β-sheet Rich Intermediates Have Larger Effects to Perturb Membranes

**Figure 6:**
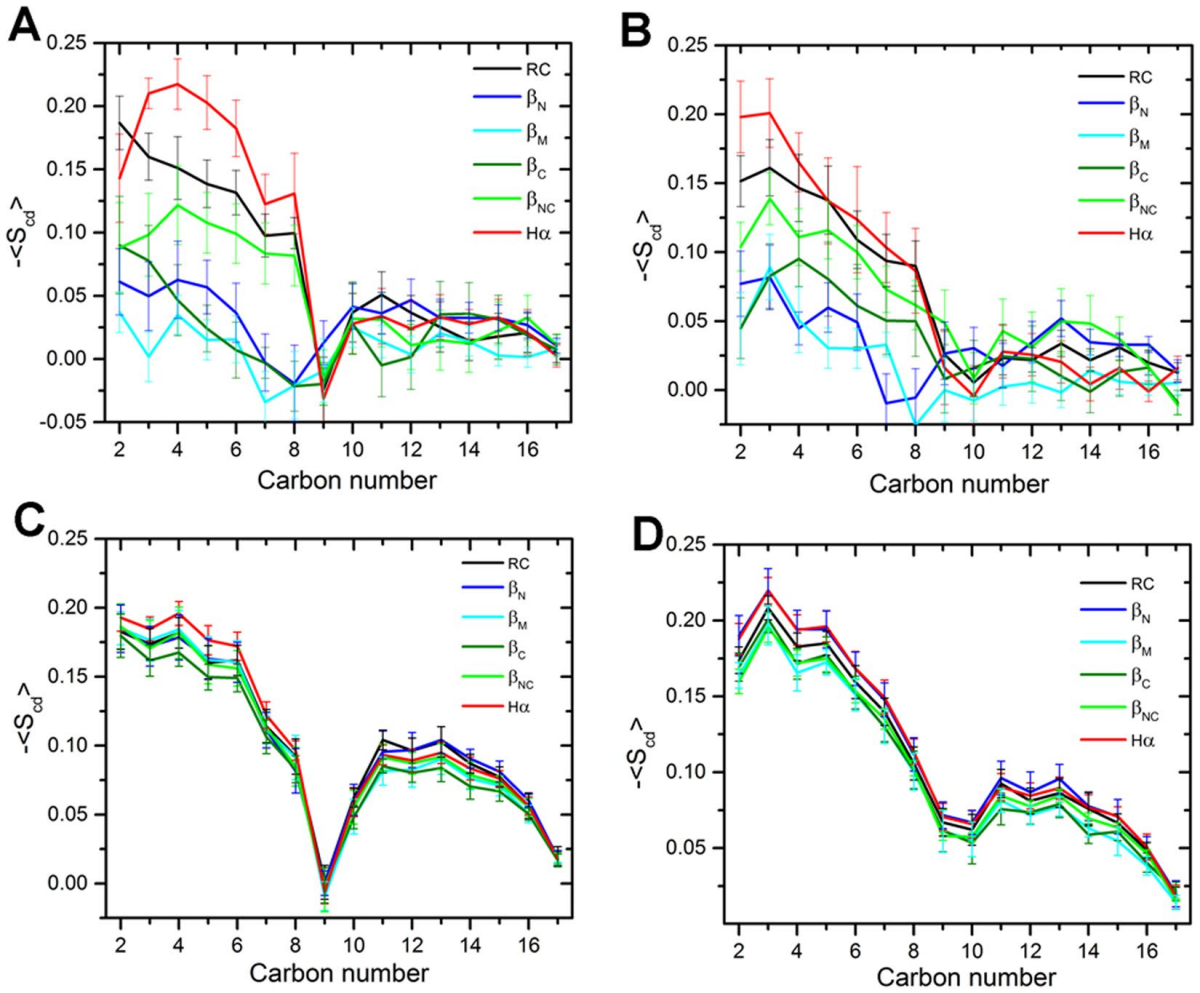
Order parameter -<*S*_*cd*_> for the acyl chains of DOPC lipids binding to different intermediates as a function of carbon atom index. (**A**) *sn*-1 and (**B**) *sn*-2 chain of the DOPC within 15 Å of the proteins. (**C**) *sn*-1 and (**D**) *sn*-2 chain of DOPC whose distances to proteins are larger than 15 Å. The angle brackets denote the average values over the last 100 ns simulation time, the error bars corresponding to the deviations of five 20-ns windows in the last 100 ns trajectories.

The disruption of β-cell membranes by small hIAPP oligomers was proposed to be responsible for the toxicity of hIAPP^22^. In this work, we performed multiple unbiased MD simulations to study the effects to the lipid bilayer by different hIAPP dimeric states obtained by the BE-Meta simulations. The six intermediates we considered here are defined as: (1) RC state, there is no α-helical and β-sheet residue on both chains (corresponding to the basin 1 in Fig. 2); (2) β_N_ state, the N-terminal strands (region 8–17) are formed in this state (basin 4 in Fig. 2); (3) β_C_ state, the C-terminal strands (region 28 to 35) are formed in this state (basin 8 in Fig. 2); (4) β_M_ state, the strands on the middle region (residue 20 to 27) are formed (basin 5 in Fig. 2); (5) β_NC_ state, both the N-terminal and C-terminal strands are formed (basin 7 in Fig. 2); (6) H state, the α-helical structure are formed on both chains. For each intermediate, two structures were randomly picked as the initial structures for the unbiased simulations. The backbone RMSDs and the C_α_ RMSFs for both chains without 2 terminal residues are monitored during the 200 ns unbiased simulations. In all the trajectories, the RMSD deviation are less than 4 Å and the C_α_ RMSF are less than 2 Å for the no-terminal residues during the 200 ns simulations except the RC state. The small RMSD and RMSF fluctuation demonstrate the stability of the intermediates detected by the BE-Meta simulations. The last 100 ns trajectories were used in the following analysis.

**Figure 7:**
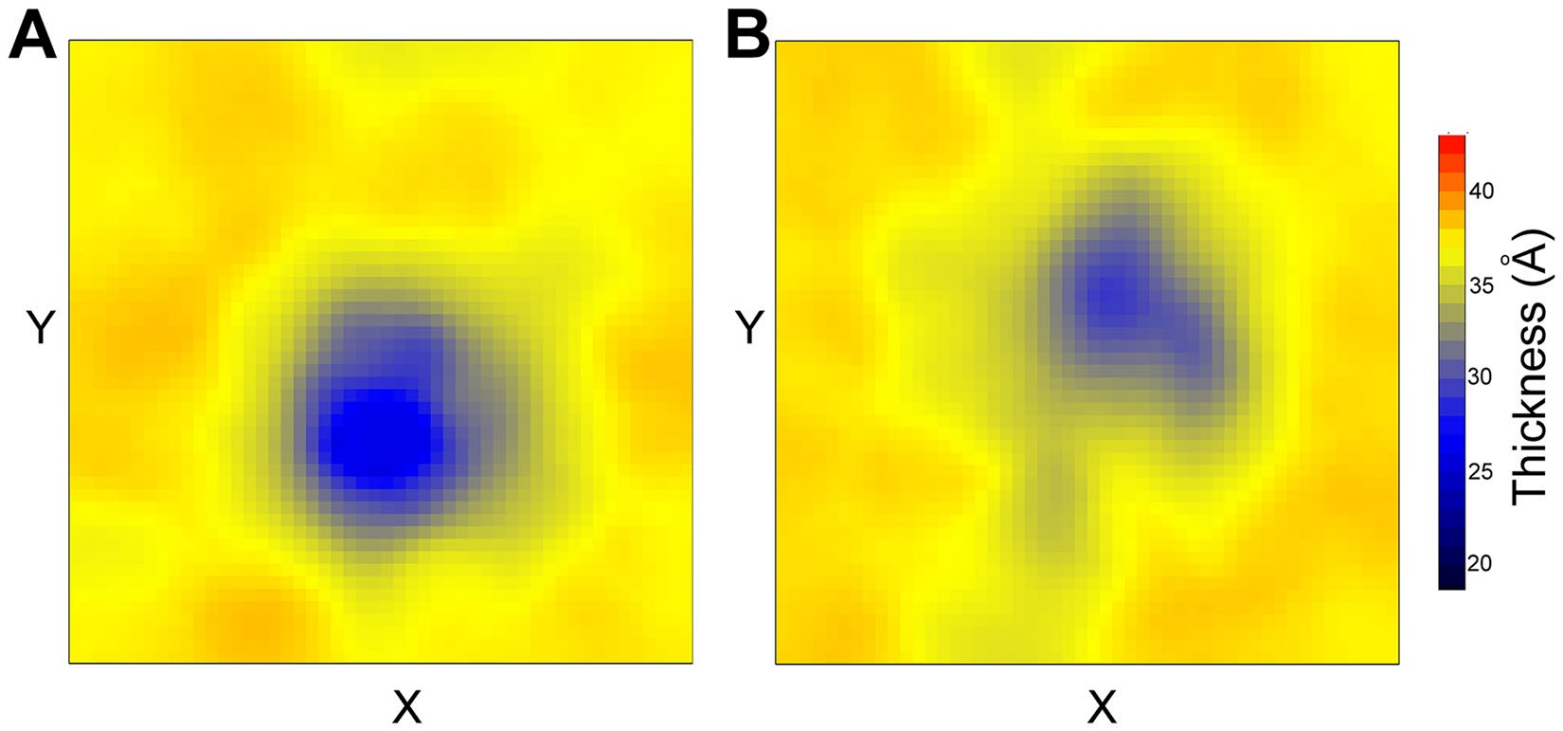
The average membrane thickness over the last 100 ns simulation time. (**A**) Membrane bounded with the β_NC_ intermediate; (**B**) Membrane bounded with H intermediate. The thicknesses are calculated the distance between the phosphate group on the opposite layer of the membrane.

The effects of dimeric hIAPP intermediates to the membrane were investigated based on the average deuterium order parameters <*S*_*cd*_> (Fig. 6), the membrane thickness (Fig. 7 and Figure S9), the area per lipid (Table S1) and the membrane curvatures (Figure S10). The deuterium order parameters of lipid acyl tails is defined as: ${S}_{cd}=\frac{1}{2}(3{\cos }^{2}\theta -1)$, where θ is the relative angle between the CD bond vector and the bilayer normal and the brackets denote averaging over molecules in simulations. *S*_*cd*_ provides a quantitative measure of the alignment of the lipid tails and related to the flexibility of the lipids, and larger -*S*_*cd*_ values are corresponding to more rigid lipid tails^78^. In order to characterize the influences of IAPP dimer to the lipid chains, we divided the lipid to two classes based on their center of mass (COM) distances to the proteins. The distances in the first kind of lipids to the COM of proteins are smaller than 15 Å, and the distances are larger than 15 Å in the second class of lipids. For each moment/snapshot we consider in the trajectory, the lipids were divided into two groups (lipids close to the dimer or far away from the dimer) based on the lipid-protein distances. Then the structural parameters (such as *S*_*cd*_) were calculated against these two groups for each conformation, respectively. And at last the mean *S*_*cd*_ values were calculated over all conformations in the trajectory. The average -*S*_*cd*_ for the *sn*-1 chain and *sn*-2 chains of the first class of DOPC were given in Fig. 6A and B. It can be seen that the - < *S*_*cd*_ > of the carbon atoms close to the lipid head-group (carbon atom index < 9) of β-sheet intermediates are obviously smaller than the disordered intermediate and helix intermediate. On the other side, the order parameters of the lipids far away from the proteins are barely influenced by the intermediates type (Fig. 6C and D). Therefore, we conclude that the β-sheet intermediates have larger effects on the lipids, causing the lipid more mobile and flexible.

The thickness analysis of the membrane also supports the above conclusions. Figure 7 and Figure S9 give the average thickness of the membranes binding with different intermediates. It can be seen that the membranes around the β-sheet intermediates are 5–10 Å thinner than the membrane around the α-helix intermediates and fully disordered intermediates. The cytoplasm leakage was proposed to account for the IAPP toxicity, the thin membrane might facilitate the cytoplasm pass through the lipid bilayers. Therefore, the β-sheet intermediates would have larger membrane perturbation ability than the helix intermediates and the disordered intermediates. Interestingly, the intermediates with only N-terminal β-sheet or C-terminal β-sheet have much more effects on the membrane thickness than fibril like structure (β_NC_ intermediate), suggesting the intermediate oligomers might have larger cell toxicity than the produced fibril structures. This observation is supported by the experimental results that the most toxic species are the oligomers than the fibril productions^18, 19^.

## Conclusions

The IAPP oligomers formed during the dimerization process and their ability to disrupt the β-cell membranes, are supposed to take charge of the hIAPP toxicity. Therefore, it is critical to understand the properties of intermediate oligomers and the early-stage aggregation process of IAPP in the membrane environment. IAPP is a good model in the study of the behaviors of the fibril formation because it shares many common features with other amyloid proteins. For example, the monomeric state of amyloid proteins like Aβ, α-synuclein and IAPP adopt transit α-helix structure in the aqueous solution, the present of membrane would stabilize the monomeric helical structure and facilitate the fibril formation. Besides, the fibrils of almost all amyloid proteins (include IAPP) share the cross-β structures. In this study, the bias-exchange metadynamics were employed to study the properties of hIAPP dimer in the lipid bilayer, the enhanced sampling technology used here could efficiently uncover the early stage of the hIAPP aggregation on the lipid bilayer environments.

The free energy profiles constructed based on our simulation data show that the 20–29 region plays important roles in the aggregation of hIAPP on the membrane environments. The formation of β-sheet structure on this region would guide the formation of toxic species. Such region undergoes a disorder-order-disorder transition in the fibril assembly process, which is similar to the process of such region in the aqueous solution^11^. It explains why the β-sheet broken mutations (A25P, S28P and S29P) on the segment 20–29 could prevent the amyloid deposition.

Unlike two comparable pathways revealed by Buchanan, *et al*. in the dimerization process of hIAPP in aqueous solution^11^, there is only one dominant pathway in the membrane environment. In membrane, the N-terminal β-sheet structure grows prior to the C-terminal β-sheet structure. The intermediates with only C-terminal β-sheet formed are more like the off-pathway intermediates to the fibril formation process. Besides, the pre-mature amyloid oligomers instead of the intermediates with the fibril structure are not the lowest free energy state in the membrane environment.

The propensity of helical residues is much less than β-sheet structure in the dimeric hIAPP, and the free energy of the intermediate with two peptide chains in α-helical structure is much higher than β-sheet intermediates. These results illustrated that it is harder to form compact α-helical IAPP dimer in the membrane than the β-strand rich intermediates in the membrane environments. Besides, the free energy profile analyses showed that the α-helical intermediates are the off-pathway states in the fibril structure assembly process of hIAPP.

We also estimated the ability of different hIAPP dimeric intermediates to perturb the membrane based on the lipid order parameters and the thickness analysis. The results indicated that the membranes with β-sheet structures are more flexible and thinner than the membranes containing α-helical IAPP or fully disordered IAPP. The decreasing of the membrane thickness could induce the leakage of cytoplasm and might be the reason of cell toxicity of hIAPP oligomers. Interestingly, the β-sheet oligomers have larger membrane disruption ability on the membrane than the mature fibril-like structures. This observation provides explanations to the greater toxicity of intermediate oligomers than the product fibrils.

In this study, we mainly focus on the structural properties of hIAPP dimer in the membrane environments. BE-Meta simulation would run over the conformation space defined by the collective variables after the convergence of the simulation. Therefore, the simulation can be started from any structure (the pre-formed fibril structure was employed in our study) and get the similar structure ensembles at the end of the simulations. In fact, lots of distinct intermediates were observed in the simulations, including fully disordered intermediates, α-helical intermediates, partially folded β-sheet intermediates and the state with fibril-like structure (Fig. 2). And the free transitions between these intermediates (Figure S5) ensure that the structural properties obtained from the simulations are irrelevant with the initial conformations.

The conformational space sampled by the BE-Meta dynamics depends on the collective variables (CVs). The CVs we selected in this study are focus on the structure properties of IAPP, the interactions between the protein and membrane might be insufficiently characterized. Employ more CVs considering the interactions between IAPP oligomers and membranes are therefore required in the further studies. Besides, the diffusions from independent monomers to the oligomers may help us to understand which kinds of the interactions dominant the fibril growth^79^. However, as we mentioned above, the thermodynamic properties of the dimeric hIAPP are not likely to be changed if individual monomers were set as the initial structure of the simulation. In addition, the surface type and membrane components have great influence on the amyloid^80^, for instance, the presentation of cholesterols in the membranes would make the phospholipids more ordered and rigid, which decreases the deposition of IAPP fibrils^15^. Besides, the gangliosides would affect the amyloid binding with membranes and the toxicity of the fibrils^81^. The study of IAPP on membranes with different kind of components would greatly extend our knowledge of the aggregation mechanism of IAPP.

Overall, based on the enhanced sampling technology and unbiased simulations, a detailed and exhaustive picture that describes the early stage of IAPP aggregation in the membrane environments was proposed in this work. Our computational results, which are agreed with recent experimental data, also provide atom-level structural morphologies of the hIAPP dimer. The hIAPP intermediates and the aggregations pathway found in this study might help to design new drugs to prevent the cytotoxicity of IAPP and treat the progression of T2D.

## Electronic supplementary material

Supporting Information_IAPP Dimer
