# Supplementary material for: Structural Properties of Human IAPP Dimer in Membrane Environment Studied by All-Atom Molecular Dynamics Simulations
Source: Sci Rep. 2017 Aug 11;7:7915. doi: 10.1038/s41598-017-08504-x (PMC5554177; doi:10.1038/s41598-017-08504-x)
Supplement: Supplementary file 1 — Supporting Information_IAPP Dimer [file 41598_2017_8504_MOESM1_ESM.pdf]

SUPPORTING INFORMATION

Structural Properties of Human IAPP Dimer in Membrane Environment Studied by All-Atom Molecular Dynamics Simulations

Na Liu[1,2], Mojie Duan[1,*], Minghui Yang[1,*]

1. Key Laboratory of Magnetic Resonance in Biological Systems, National Center for Magnetic Resonance in Wuhan, State Key laboratory of magnetic Resonance and Atomic and Molecular Physics, Wuhan Institute of physics and Mathematics, Chinese Academy of Sciences, Wuhan 430071, China
2. University of Chinese Academy of Sciences, Beijing 100049, People's Republic of China

*Correspondence to:

mjduan@wipm.ac.cn; yangmh@wipm.ac.cn

Initial model of IAPP dimer in the membrane. In this study, the initial dimer model of hIAPP was taken from a solid-NMR pentamer, and a 50-ns NPT-simulation were performed on the structure in the aqueous solution. There is no large structure change on the dimer during the simulation (the heavy-atom RMSD of core-region is about 2.2 A between the first and the last snapshots), therefore, the last snapshot in the 50-ns simulation was selected to build the membrane-IAPP dimer model. Then, another 50-ns NPT regular MD simulation was performed on the membrane with IAPP dimer to test the stability of the system. The results show that the scaffold of the dimer remained stable, and the Cα RMSD between the protein conformations in the simulation and the NMR structure are around 3Å (Figure S2A). Based on this, we believed that the dimer structure used here is a reliable structure model.

The initially orientation and position of hIAPP dimer in the membranes were set according to the SFG spectrum results, i.e. the initial tilt angle between the β-strand of residue 8 to 16 and the membrane interface was set to be 48°. Although the membrane components are different with the experiment (pure DPPG lipids were used in the SFG spectrum experiment and mixture DOPC/DOPS lipids were used in in our simulations), the tilt angles in the 50-ns regular simulation fluctuated around the value of 50°, which is consistent with the experimental value. To further demonstrate the reliability of the tilt angle, a fast sampling approach (highly mobile membrane-mimetic, HMMM model)[1] was employed to character the tilt of angle of IAPP dimer in the DOPC/DOPS membrane by a 50 ns simulation. The results showed that the system can be stabilized in the tilt angle around 40° (Figure S2B). All the above results demonstrated that the initial structure and orientation of IAPP dimer we used are reasonable.

BE-Meta simulation vs Replica-exchange MD (REMD). For temperature based REMD (tREMD), multiple replicas run simultaneously at different temperatures and the adjacent replicas randomly exchanged based on the Metropolis criterion. It is possible to overcome the energetic barriers after exchange the system from low-temperature replica to high-temperature replica. For metadynamics, the system can escape from the energy minima by accumulating history-dependent Gaussian potential on specific collective variables (CVs), and the free energy surface on these CVs can be recovered by the total bias potential energy

added after the convergence of the simulations. However, it is very hard to reach convergence for the systems with more than three CVs. The bias-exchange metadynamics (BE-Meta) combine replica exchange and metadynamics, which perform potential bias on a large number of CVs and allow conformations exchanged between these CVs. In this way, the energetic barriers can be crossed in a short simulation time.

Collective Variables Used in BE-Meta. In our simulation, eight replicas were employed to explore hIAPP dimerization process, where two replicas were not biased by time-dependent potential but allowed to exchange conformations with the others (the *neutral replicas*) the rest six replicas were biased on different six CVs. The acceptance ratio of the exchange is:

$$P = \min\left\{1, \exp\left[\frac{1}{T}\left(V_G^a(x^a,t) + V_G^b(x^b,t) - V_G^a(x^b,t) - V_G^b(x^a,t)\right)\right]\right\}$$

where $\boldsymbol{x^a}, \boldsymbol{x^b}$ are the coordinates of conformations in replica *a* and *b*. $V_G^{a(b)}(x,t)$ is the metadynamics potential acting on the replica *a* or *b*. Conformation swaps would happen when P=1.

The six biased CVs are:

CV1. *Q parameter for residues 8 to 16 and residues 27-35*, used to count the number of contacts between two hIAPP chains, defined as:

$$Q = \frac{1}{N_{res}} \sum_{i,j} C_{ij}$$

With

$$C_{ij} = \frac{1 - \left(\frac{r_{ij} - d_0}{r_0}\right)^n}{1 - \left(\frac{r_{ij} - d_0}{r_0}\right)^m} \qquad (2)$$

where N_{res} is the considered residue number, here N_{res}=18, r_{ij} is the distance between atoms in different chains, where n=6, m=12, d_0=0.3 and r_0=0.212, the sum of equation 2 runs over backbone O and N atoms in residues 8 to 16 and residues 27 to 35 of two monomers.

CV2. β-score *for residues 8 to 16*, this variable counts the number of pairs of 3-residue segments in two monomers that are similar to ideal parallel β-sheet. This CV is calculated by:

$$\beta - score = \sum_i \frac{1-\left(\frac{r_i}{0.08}\right)^8}{1-\left(\frac{r_i}{0.08}\right)^{12}} \quad (3)$$

CV3. β-*score for residues 27 to 35*, this variable has the same definition as CV2, but for residues 27 to 35. It characterizes all the possible parallel β-sheet formed at the C-terminal of two monomers.

CV4. β-*score for residues 18 to 28*, same definition as CV2 and CV3, it counts how many pairs of 3-residue blocks formed parallel β-sheets between residue 18 and residue 28 in two monomers.

CV5. α-*score for residues 8 to 28 in the first chain of hIAPP dimer,* this CV was used to measure the number of 6-residue segments that form α-helix, the calculation of this CV is similar to CV2.

CV6. α-score *for residues 8 to 28 in the second chain of hIAPP dimer,* this CV has the same definition as CV5, but for the other chain of hIAPP dimer.

1 Ohkubo, Y. Z., Pogorelov, T. V., Arcario, M. J., Christensen, G. A. & Tajkhorshid, E. Accelerating membrane insertion of peripheral proteins with a novel membrane mimetic model. ***Biophys J*** 102:2130-2139(2012).

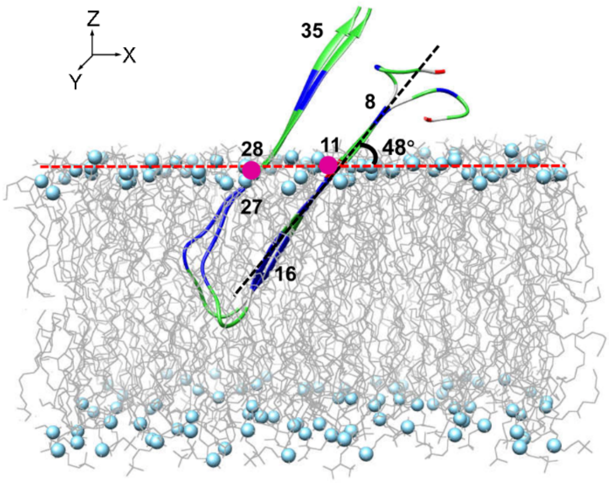

Figure S1. The initial structure model of dimeric IAPP in the membrane. The β-strands of initial IAPP dimeric structure are located on the residues 8 to 16 and 27 to 35, which are represent by flat arrow. The Cα-Cα vector (dark dashed line) between residue 9 and 15 for the N-terminal β-strand were used to define the orientation between IAPP and the membrane (the tilt angle is 48°). The membrane-water interface (red dashed line) was defined by the phosphorus atoms of the lipids, which are shown as cyan spheres. The residues 11 and 28 on the membrane interface are labeled and shown by magenta cycles. The residues are colored by blue for hydrophobic, green for polar and red for positive charged. The initial Z-direction distance between the centers of masses of hIAPP dimer with the upper membrane is 6.4 Å.

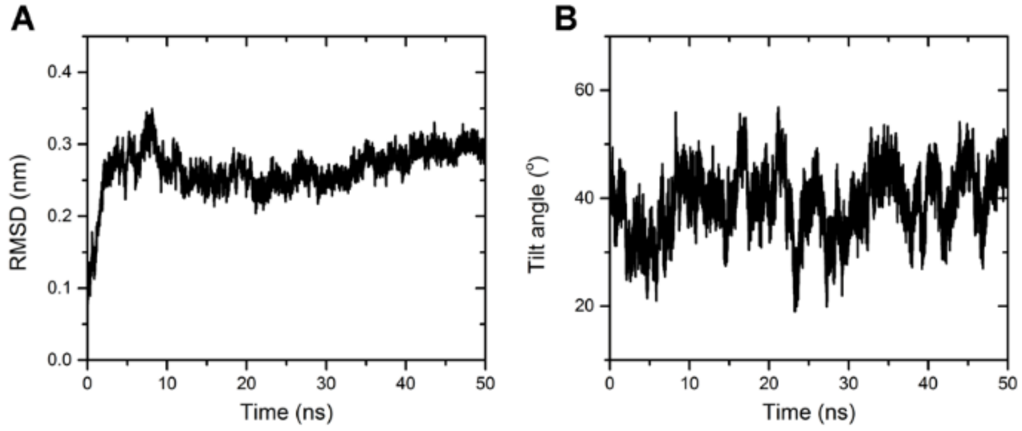

Figure S2. Tests on the initial structure model. (A) Cα RMSD of IAPP dimer in the membrane as a function of simulation time; (B) The tilt angle of IAPP dimer in the membrane as a function of simulation time with the HMMM model.

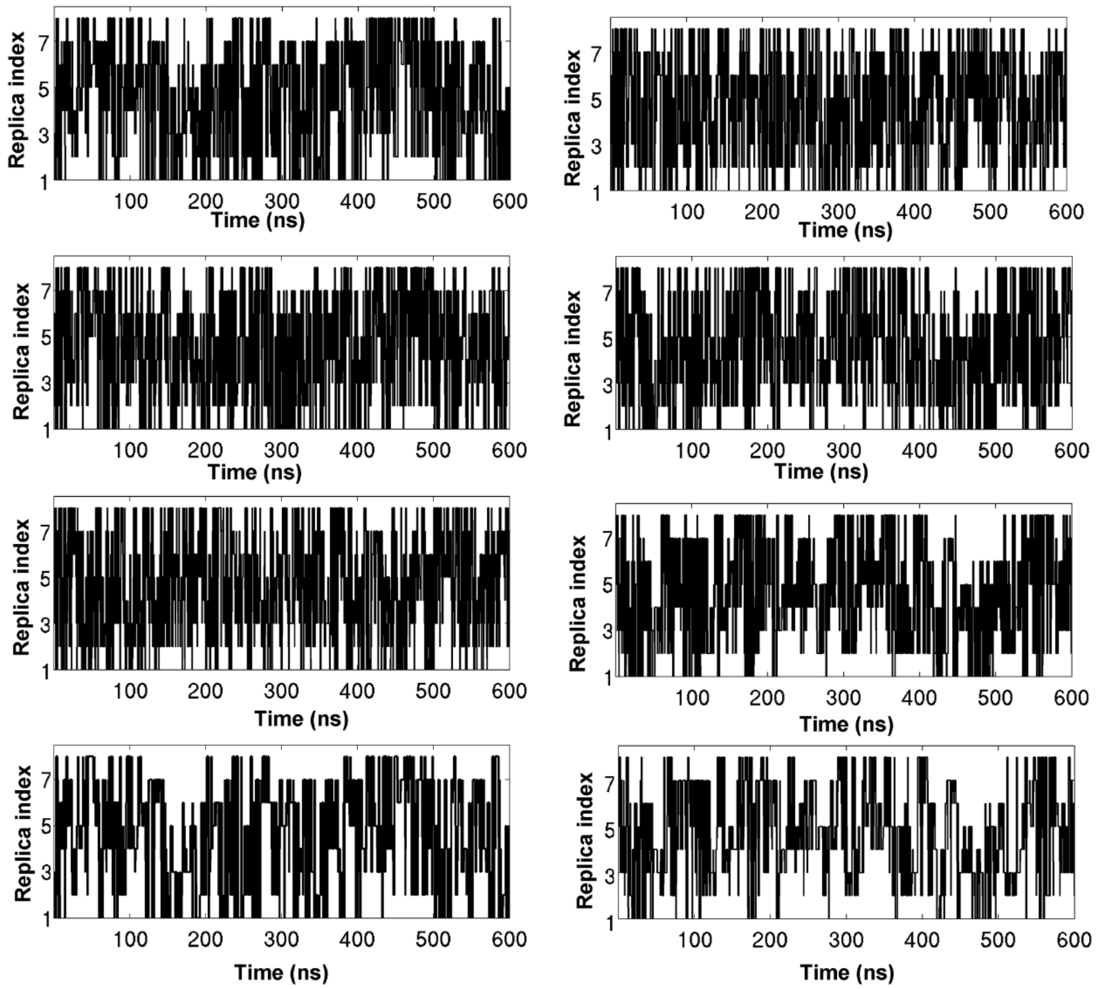

Figure S3. The exchange between 8 replicas as a function of simulation time.

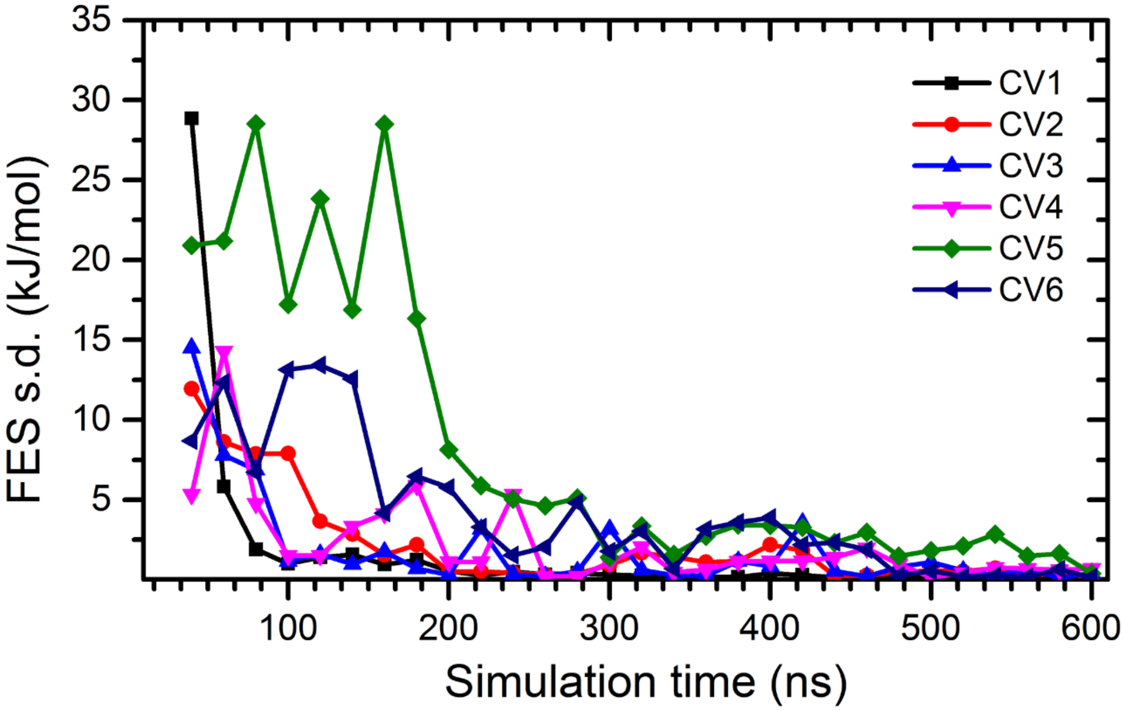

Figure S4. Free energy change as a function of simulation time. Free energy change from time *t*-*δt* to *t*, here *δt*=20 ns.

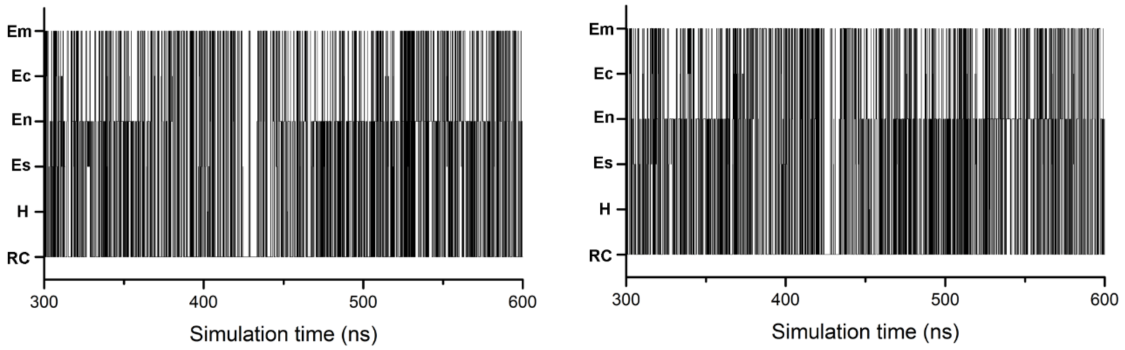

Figure S5. Transitions between different intermediates along the simulation trajectories of the last 300 ns of two neutral replicas. Es: intermediate with more than six β-sheet residues on both N-terminal (residue 8-17) and C-terminal region (residue 28-35); En: intermediate with more than six β-sheet residues on the N-terminal region; Ec: intermediate with more than six β-sheet residues on C-terminal region; Em: intermediate with more than six β-sheet residues on the middle region (residue 18-27); H: intermediate with more than eight α-helical residues on any chain; RC: including all other conformations.

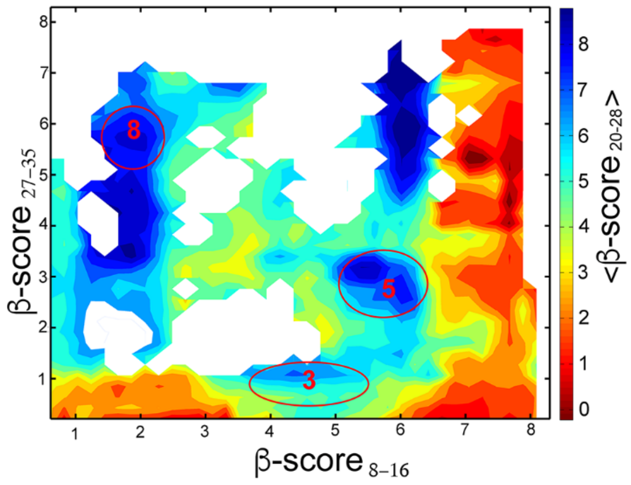

Figure S6. Average length of β-sheet structure in the region 20 to 27 was plot as a function of the order parameters of β-score$_{8\text{-}16}$ and β-score$_{27\text{-}35}$. The regions labeled by the number are corresponding the locations of intermediate 3, 5, and 8.

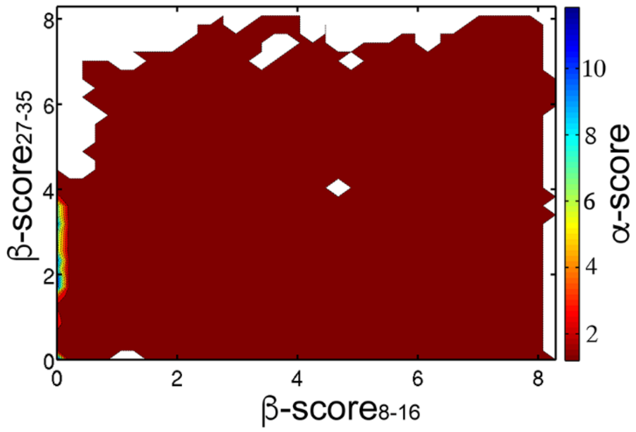

Figure S7. The average helix length as a function of the order parameters of β-score$_{8-16}$ and β-score$_{27-35}$.

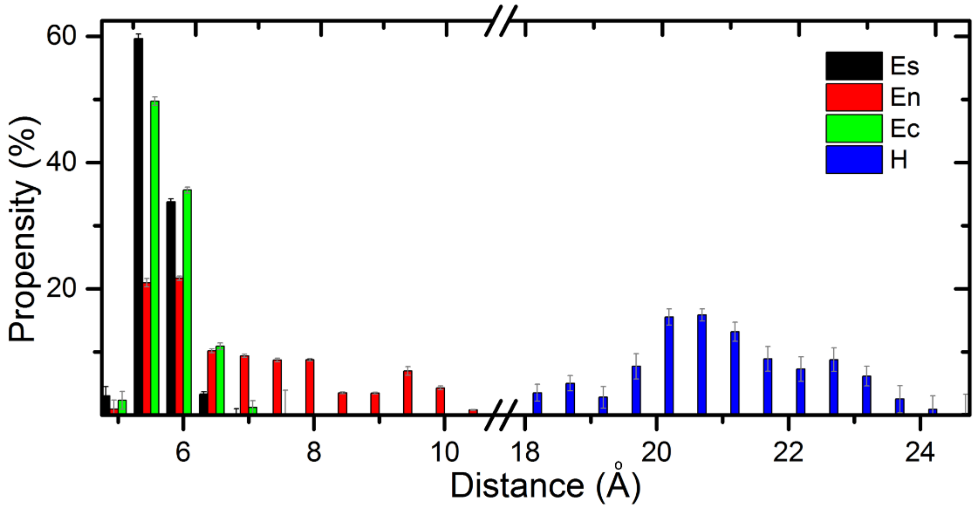

Figure S8. Distribution of distances between two chains in different intermediates.

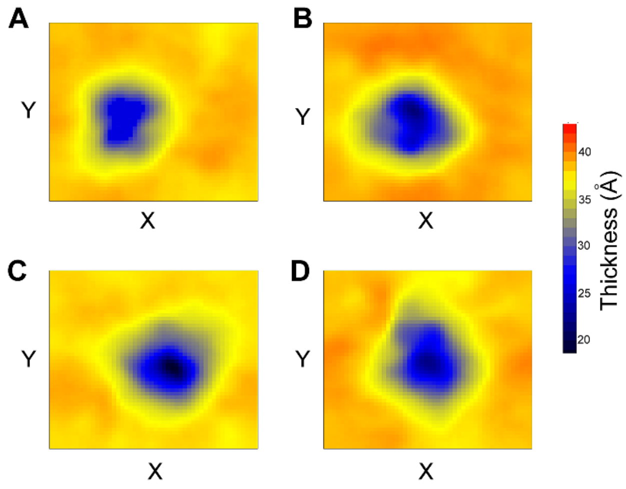

Figure S9. The thicknesses of lipid bilayers bounded with different intermediates. (A) RC; (B) β_N; (C) β_M; (D) β_C.

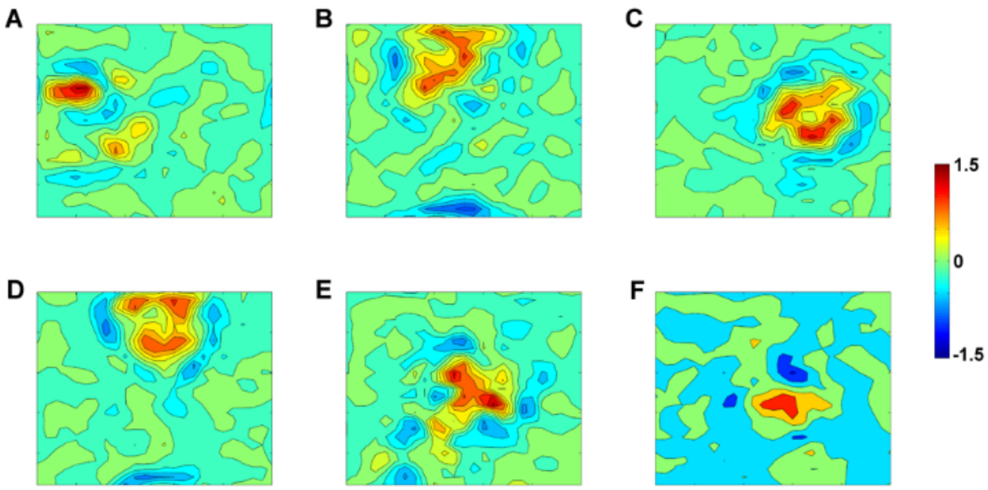

Figure S10. The mean curvature of the upper layer of the membranes bounded with different intermediates. (A) RC; (B) β_N; (C) β_M; (D) β_{NC}; (E) β_C; (F) α-helical intermediate.

Table S1. The area per lipid (APL) for the membranes bounded with different intermediates.

	RC	β_N	β_M	β_{NC}	β_C	α-helix
APL (Å^2)	60.8±1.3	62.5±1.3	63.0±1.4	61.6±1.4	62.7±1.8	58.0±1.5
